# Complete chloroplast genome sequence of the medicinal plant *Actaea cimicifuga* L. (Ranunculaceae)

**DOI:** 10.1080/23802359.2026.2677955

**Published:** 2026-05-25

**Authors:** Kesong Li, Han Zhang, Xiang Gao, Haobin Wang, Ruihong Gui, Yaqiong Li

**Affiliations:** College of Traditional Chinese Medicine, Yunnan University of Chinese Medicine, Kunming, China

**Keywords:** *Actaea cimicifuga*, complete chloroplast genome, Ranunculaceae, medicinal plant

## Abstract

*Actaea cimicifuga* L. (syn. *Cimicifuga foetida* L.) is an important medicinal plant widely used in China. In this study, the complete chloroplast genome of *A. cimicifuga* was characterized and assembled. The complete chloroplast genome is 159,761 bp in length and exhibits a typical tetrad structure, consisting of a large single-copy region (88,805 bp) and a small-single copy region (17,838 bp), separated by two inverted repeat regions of 26,559 bp each. A total of 129 unique genes were annotated, including 84 protein-coding genes, 8 rRNA genes, and 37 tRNA genes. Fourteen genes containing introns were identified in the chloroplast genome of *A. cimicifuga.* Among these, two genes (*ycf*3 and *clp*P) contain two introns, whereas the remaining genes contain a single intron. Phylogenetic analysis based on maximum likelihood using 20 complete chloroplast genomes showed that *A. cimicifuga* is closely related to other species within the *Actaea* genus. The chloroplast genome of *A. cimicifuga* provides valuable genetic information.

## Introduction

**Figure 1.**
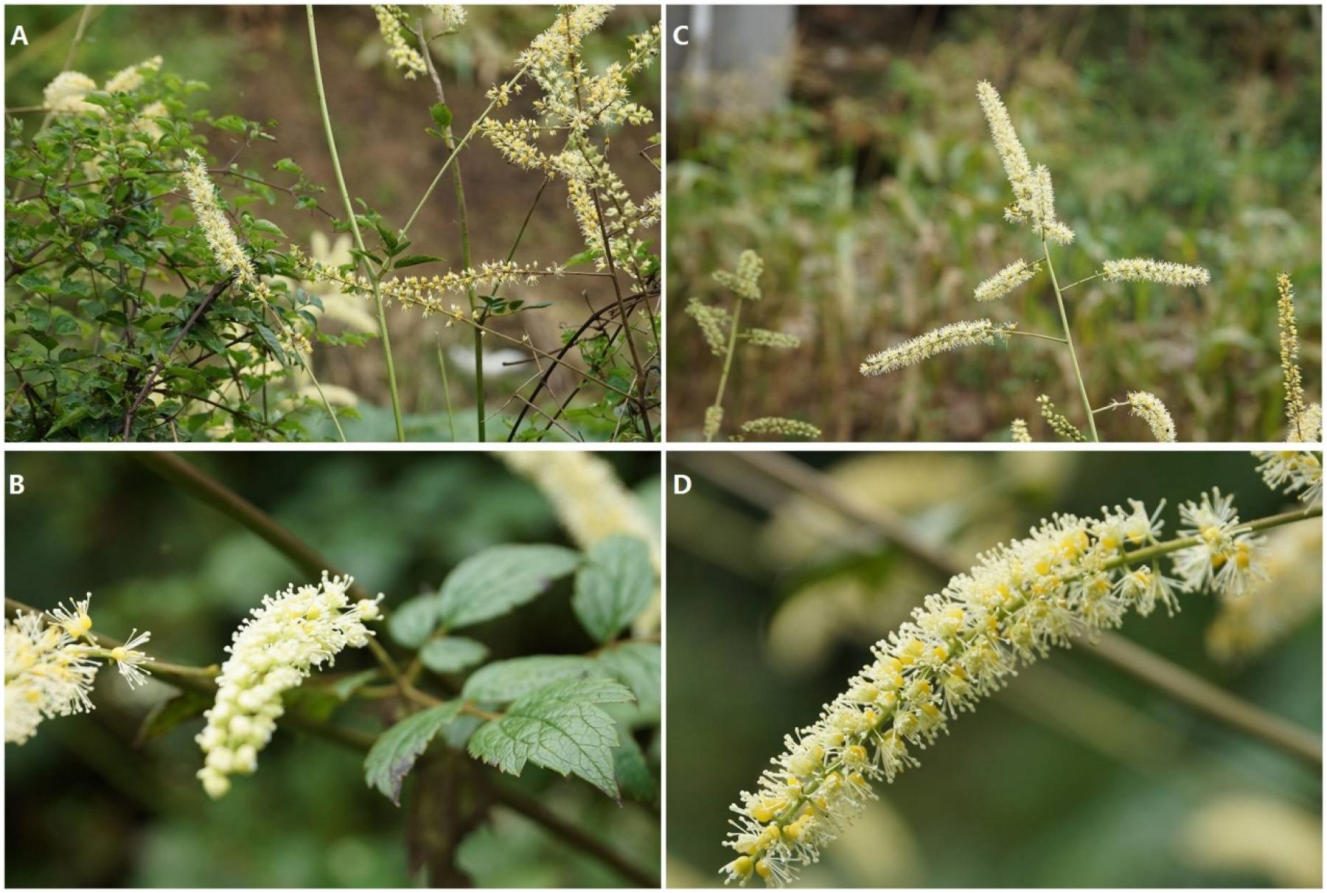
Photographic documentation of *A. cimicifuga* (photographs taken by congwei yang in deqing, China). (A) Habitat and whole plant morphology. (B) Inflorescence and leaf morphology. (C) Habitat showing multiple inflorescences. (D) Detailed view of a single inflorescence. The stems are erect, and the leaflets are oval to lanceolate with coarsely serrated margins. The leaves are green, their back is gray-green, and both sides are pubescent. The flowers are small, yellow and white, arranged in panicles, and born at the apices of the branches.

*Actaea cimicifuga* L. (syn. *Cimicifuga foetida* L.) is a perennial herb of the genus *Actaea* in the family Ranunculaceae (Figure 1). *C. foetida* L. was first described in Syst. Nat. ed. 12,659 in 1767 (Editorial Committee of Flora of China CAS 1979). *A. cimicifuga* L. (Sp. P1.504.1753) and *C. foetida* L. are considered to represent the same species, as recorded in ‘Qinling Flora’ in China (Editorial Committee of Flora of China CAS 1979; Editorial Committee of Flora of Qinling Mountains CAS 1985). *A. cimicifuga* L. is listed in the first part of the 2020 edition of the Chinese Pharmacopeia as one of the source plants of the cosinense. The genus *Actaea* (Ranunculaceae) comprises approximately 18 species distributed across temperate regions of the Northern Hemisphere (Editorial Committee of Flora of China CAS 1979), of which eight species occur in China.

The genus *Actaea* comprises perennial species characterized by erect stems and stout rhizomes. In China, approximately eight *Actaea* spp. are used as medicinal plants to treat various types of pain and rheumatoid arthritis (RA) (He et al. 2006). *A. cimicifuga* is a medicinal perennial herb distributed in western, southwestern, and northwestern China at altitudes of 1,700–2,300 m. Phytochemical studies have shown that this species contains various bioactive compounds, including triterpenoid saponins, phenolic acids, chromones, alkaloids, saccharides, and sterols (Chen et al. 2014; Shi et al. 2018). These compounds have been associated with multiple pharmacological activities, such as anti-proliferative (Ahn et al. 2013), anti-inflammatory (Li and Yu 2006), anti-osteoporotic (Li et al. 2007; Nian et al. 2010), and anti-angiogenic effects, as well as alleviation of menopausal symptoms (Wuttke et al. 2003). *A. cimicifuga*, commonly known as ‘Shengma’ in traditional Chinese medicine (Li et al. 1994), is one of the most important medicinal herbs and has long been used by various ethnic groups in China to treat rheumatoid arthritis.

The natural habitat and population of *A. cimicifuga* have been greatly reduced by human activity (Shen et al. 2013). Chloroplast (cp) genomes are widely used in phylogenetic studies, DNA barcoding, analyses of genome evolution, and species conservation (Malé et al. 2014). The chloroplast genome and its key genes have highly conserved structures and moderate mutation rates, making it a valuable resource for evolutionary studies. In addition, chloroplasts are instrumental in the biosynthesis and metabolic regulation of plant secondary metabolites (Wang et al. 2024). Many bioactive compounds found in traditional medicinal plants are derived from secondary metabolites synthesized in chloroplasts, suggesting a close relationship between chloroplast evolution and their medicinal value (Gomez-Casati et al. 2022).

Although *A. cimicifuga* possesses significant pharmacological properties and medicinal potential, its genetic basis has not been extensively studied, especially due to the limited availability of chloroplast genome data. Therefore, in this study, we sequenced and characterized the complete chloroplast genome of *A. cimicifuga* to clarify its phylogenetic relationships. This work provides abundant information for future studies on the phylogeny and genetic diversity of *Actaea* spp. and on *A. cimicifuga*.

## Materials and methods

Fresh leaves of *A. cimicifuga* leaves were collected from Deqing County (28°52.568′ N; 98°96.082′ E; altitude: 3480 m), Yunnan Province, China. The collection of plant materials complied with the wild plant protection regulations of the People’s Republic of China, and permission was obtained from the local Forestry and the Grassland Bureau of Yunnan Province. A voucher specimen of *A. cimicifuga* was deposited in the Herbarium of Yunnan College of Traditional Chinese Medicine (Certificate No.: AC20220828; Li Yaqiong; liyaqiong6@ 126.com). Professor Li Yaqiong identified the specimen, and total genomic DNA was extracted using the CTAB method (Li et al. 2013). DNA quality and concentration were assessed using agarose gel electrophoresis and spectrophotometry. High-quality DNA was fragmented by ultrasonication using a Covaris instrument. Following end repair, adapter ligation, and size selection, DNA fragments of approximately 270 bp was selected. The fragments were amplified using PCR, and the amplified products were used to construct a sequencing library after the removal of primer dimers. The complete chloroplast genome was sequenced using the Illumina HiSeq X-TEN platform (Illumina, San Diego, CA, USA). Raw reads were assembled using SPAdes v3.9.1 (Bankevich et al. 2012), with k-mer lengths sizes ranging from 21 to 105 bp. The assembly was further refined using Geneious v9.1.4 (Kearse et al. 2012) and Bandage v0.8.1 (Wick et al. 2015) under reference-guided conditions. Genome annotation was performed using GeSeq (https://chlorobox.mpimp-golm.mp g.de/geseq.html) (Tillich et al. 2017), with *A. heracleifolia* (GenBank accession number: MH539824) as the reference, and gene boundaries were manually corrected. Phylogenetic analysis was conducted using RAxML (Stamatakis 2014) to construct a maximum likelihood (ML) tree based on 20 complete chloroplast genomes to determine the phylogenetic position of *A. cimicifuga*.

## Results

**Figure 2.**
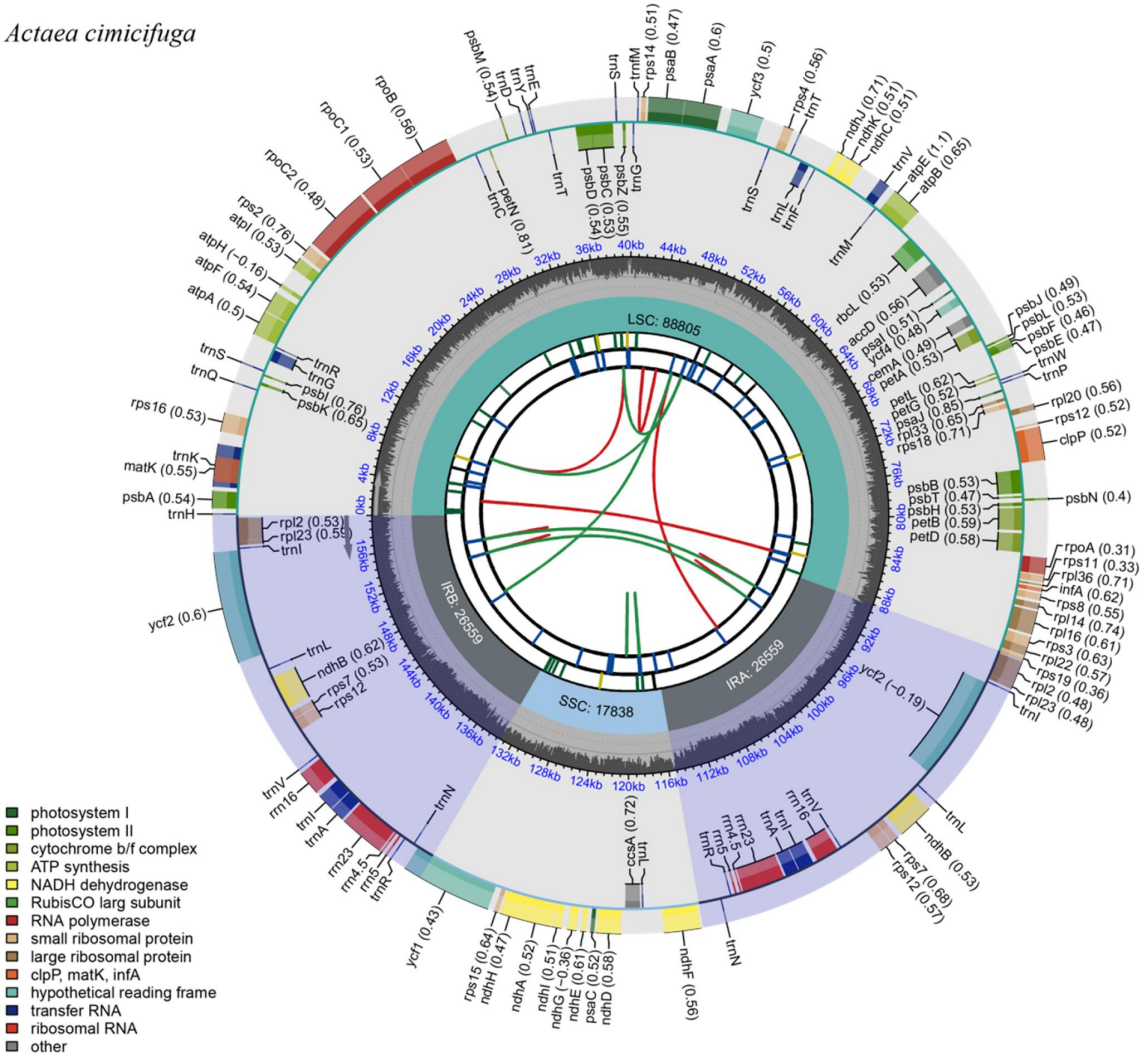
Circular map of the chloroplast genome of *actaea cimicifuga*. The complete genome is 159,761 bp in length and comprises a large single-copy (LSC, 88,805 bp) region, a small single-copy (SSC, 17,838 bp) region, and two inverted repeat regions (IRa and IRb; 26,559 bp each). The outermost circle indicates gene names and their positions, with nucleotide similarity to *A. heracleifolia* (MH539824) shown in parentheses. Genes are color-coded according to functional categories: green (photosystem-related genes), yellow (NADH dehydrogenase genes), red (*rbcL*), brown (RNA polymerase and ribosomal protein), blue (tRNA and rRNA), and grey (genes of unknown function). The Middle circle represents repetitive sequences (red: forward repeats, green: palindromic repeats) and intron-containing regions. The inner ring highlights the boundaries of the IR, LSC, and SSC regions, shown in grey, cyan, and light blue, respectively. The innermost scale indicates the physical positions along the genome (kb).

**Figure 3.**
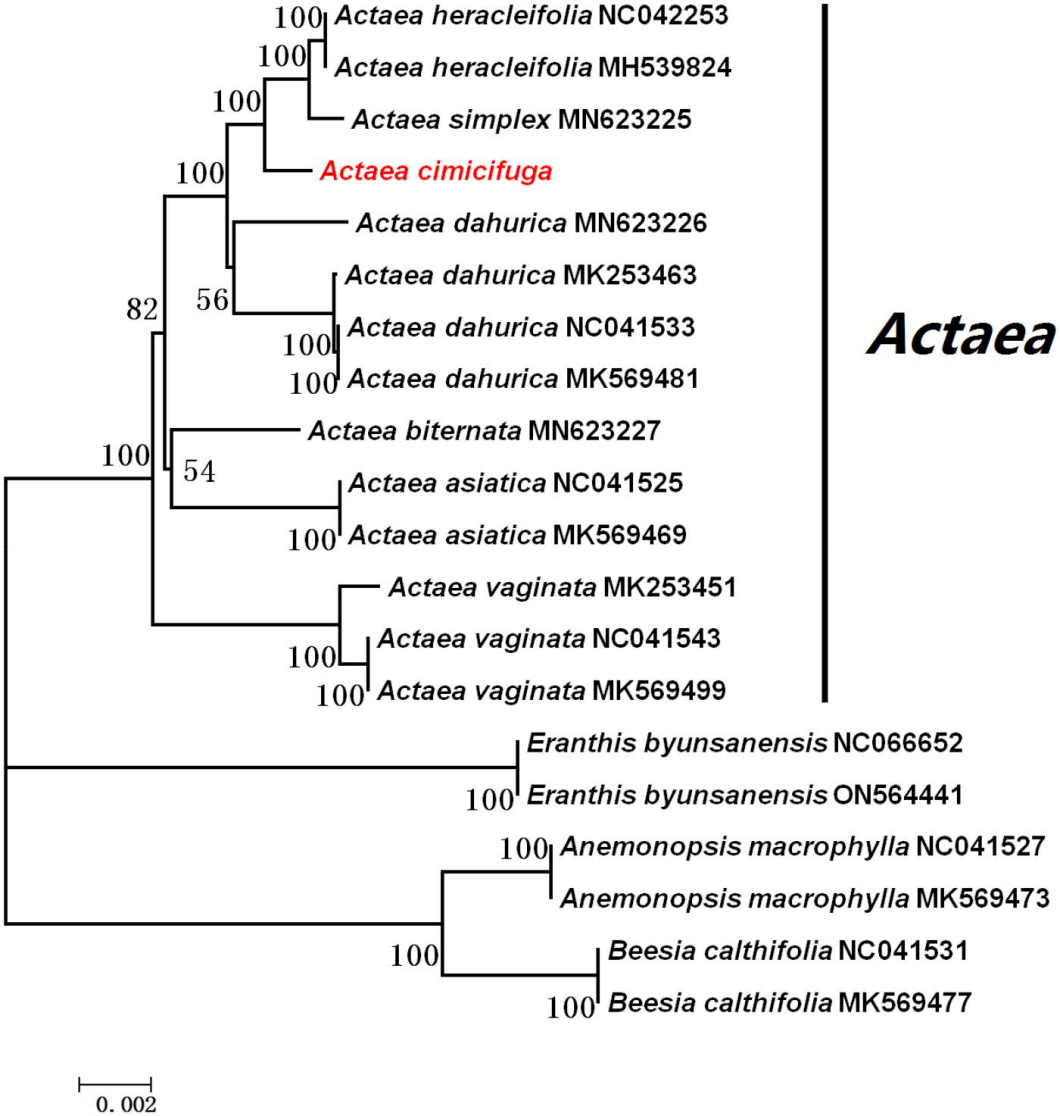
Maximum likelihood (ML) tree was constructed using 20 complete chloroplast genome sequences, with 1,000 bootstrap replicates. The analyzed sequences include: A. heracleifolia NC 042253, A. simplex MN 623225, A. dahurica MN 623226, A. biternata MN 623227, A. vaginata NC 041543 (Park et al. 2020b), A. heracleifolia MH 539824, anemonopsis macrophylla MK569473, beesia calthifolia MK569477 (Zhang et al. 2023), A. dahurica MK 253463, A. vaginata MK 253451 (He et al. 2019), A. dahurica MK 569481, A. asiatica MK 569469, A. vaginata MK 569499, eranthis byunsanensis on 564441 (Park et al. 2023), and anemonopsis macrophylla NC 041527 (Zhang et al. 2021), and four other complete gene sequences obtained from genbank, A. dahurica NC 041533 (A. dahurica voucher ZW12-003 chloroplast, complete genome), A. asiatica NC 041525 (A. asiatica voucher ZR11-020 chloroplast, complete genome), E. byunsanensis NC 066652 (E. byunsanensis chloroplast, complete genome), and B. calthifolia NC 041531 (B. calthifolia voucher ZR11-022 chloroplast, complete genome).

The complete chloroplast genome of *A. cimicifuga* exhibits the typical quadripartite structure observed in most angiosperms (Figure 2). The genome is 159,761 bp in length, with a large single-copy (LSC) region of 88,805 bp and small single-copy (SSC) region of 17,838 bp, separated by a pair of inverted repeat (IR) regions of 26,559 bp each. The nucleotide composition of the cp genome is uneven (30.7% A, 19.4% C, 18.7% G, and 31.2% T). The overall GC and AT contents are 38.1% and 61.9%, respectively. The GC content in the IR regions (43.1%) is higher than that of the SSC (32.3%) and LSC (36.3%) regions. Overall, 129 unique genes were annotated, including 84 protein-coding genes, 8 rRNA genes, and 37 tRNA genes. Among these, four rRNA genes, seven tRNA genes, and five protein-encoding genes are duplicated within the IR regions. In addition, 14 genes containing introns were identified in the chloroplast genome of *A. cimicifuga*. Of these, two genes (*ycf*3 and *clp*P) contain two introns, whereas the remaining genes each contain a single intron. Phylogenetic analysis was performed using 20 complete chloroplast genomes to construct the ML tree with 1,000 bootstrap replicates. The resulting phylogenetic tree showed that *A. cimicifuga* forms a well-supported monophyletic clade with other *Actaea* spp., with a bootstrap support value of 100% (Figure 3).

## Discussion and conclusions

*A. cimicifuga* is an important medicinal plant traditionally used in Chinese Medicine for its effects in clearing heat, detoxification, and elevating yang qi. It has been used to treat conditions such as toothache, sore throat, and incomplete rash eruption. Owing to its substantial medicinal value, this species has attracted considerable research interest. In the present study, to date, this is the first time *A. cimicifuga* undergoes chloroplast genome assembly and characterization. The genome exhibits a typical tetrad structure and is 159,761 bp in length, containing 129 annotated genes, with an overall GC content of 38.1%. Comparative analysis indicated that the chloroplast genome of *A. cimicifuga* is highly conserved in terms of genome structure, GC content, gene composition, and intron distribution when compared with other *Actaea* spp., including *A. heracleifolia*, *A. simplex*, and *A. dahurica*. Phylogenetic analysis demonstrated that *A. cimicifuga* is most closely related to *A. heracleifolia* and *A. simplex*, with strong bootstrap support (100%), whereas its relationship with *A. dahurica* is comparatively more distant (Park et al. 2018, 2020a). These data revealed the high conservation of chloroplast genomes within the genus *Actaea* and provides valuable genetic resources for species classification, molecular identification, and evolutionary research.

The chloroplast genome can be used as a reliable basis for species identification and phylogenetic analysis (Chen et al. 2022). Phylogenetic reconstruction provides important reference information for understanding species evolution. In this study, an ML phylogenetic tree was constructed using RAxML (Stamatakis 2014). The nucleotide substitution model was GTR + G + I, with 1,000 bootstrap replicates. *Anemonopsis macrophylla* (MK569473, NC041527), *Beesia calthifolia* (MK569477, NC041531), and *Eranthis byunsanensis* (ON564441, NC066652) were used as outgroups. The phylogenetic tree was constructed based on the chloroplast genomes of eight *Actaea* spp. and six representatives of Ranunculaceae. *A. heracleifolia* and *A. simplex* clustered together with strong bootstrap support (100%) and subsequently formed a clade with *A. cimicifuga. A. dahurica* was also grouped within this larger *Actaea* clade. *A. biternata*, *A. vaginata*, and *A. macrophylla* formed a separate clade, indicating a relatively conserved phylogenetic structure within the genus *Actaea*, conforming to the law that ‘many plant families and genera can be clustered into one branch on the phylogenetic tree’. The outgroup species *Anemonopsis macrophylla* and *Beesia calthifolia* clustered together in a single clade and were subsequently grouped with *E. byunsanensis* and the genus *Actaea*, forming distinct phylogenetic branches. These results further demonstrated that the chloroplast genome sequence can be used as a scientific basis for identifying genetic relationships among species. This study provides a preliminary analysis of *A. cimicifuga* based on chloroplast genome data. However, a more comprehensive understanding of species evolution and development will require integration of additional data sources, such as transcriptomic and whole-genome datasets.

Overall, this work enhances current knowledge of the genetic information of *A. cimicifuga* and provides a theoretical basis for the development of molecular markers, phylogeographic studies, and the reconstruction of phylogenetic relationships within *Actaea* in the family Ranunculaceae. These findings contribute to the growing body of research on *A. cimicifuga* and provide a theoretical basis for further research.

## Supplementary Material

The supplementary figures.docx

## Data Availability

The *Actaea cimicifuga* complete plastome sequence of Actaea cimicifuga has been stored in GenBank with the accession number OQ587922. All information can be found on the following website (https://www.ncbi.nlm.nih.gov/). The associated BioProject, SRA, and Bio-Sample numbers are PRJNA947379, SRR23936236, and SAMN33849037, respectively. Data were collected without violating the protection of human subjects or other valid ethical, privacy, or security concerns.
