# Supplementary material for: Complete chloroplast genome sequence of the medicinal plant Actaea cimicifuga L. (Ranunculaceae)
Source: Mitochondrial DNA B Resour. 2026 May 25;11(6):769–74. doi: 10.1080/23802359.2026.2677955 (PMC13202692; doi:10.1080/23802359.2026.2677955)
Supplement: The supplementary figures.docx [file TMDN_A_2677955_SM1411.docx]

**The supplementary figures**

1. **coverage depth plot**

**
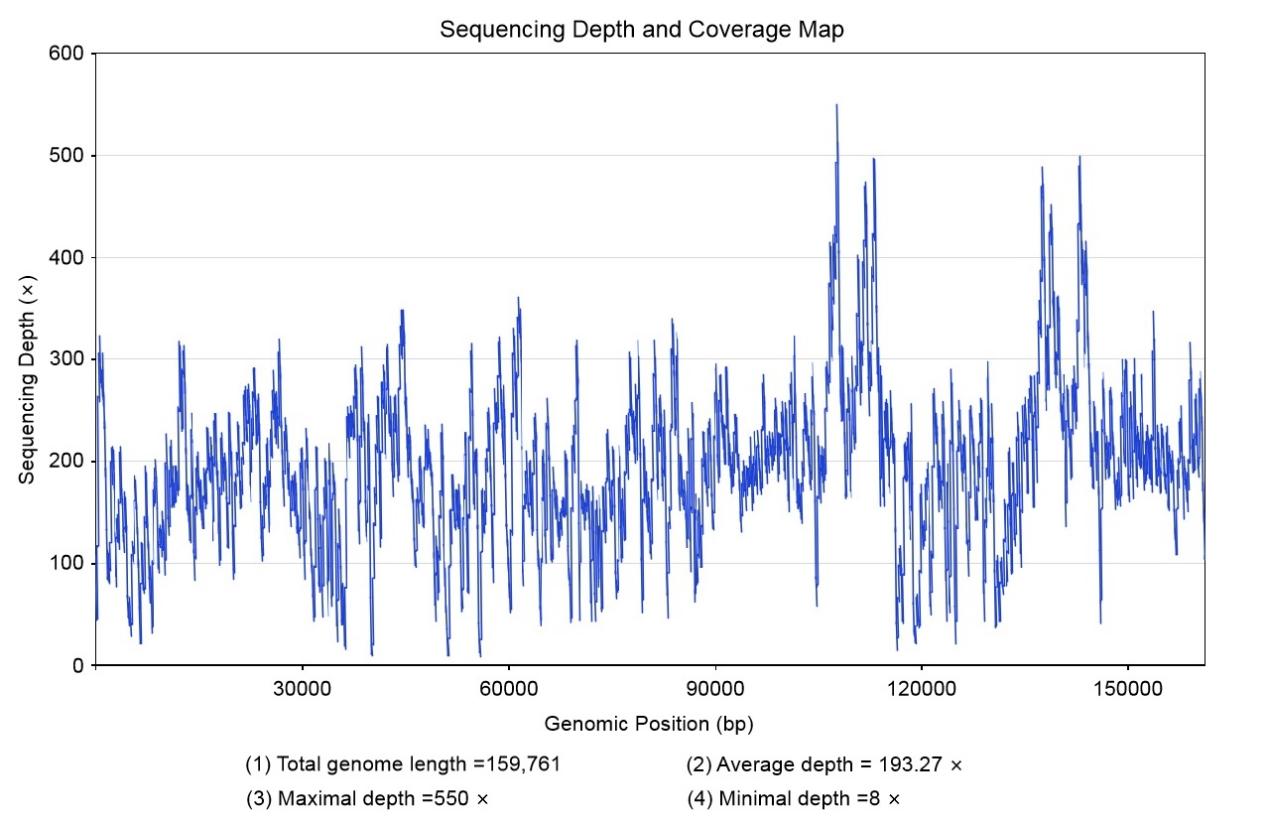
**

**Figure 1.** The figure shows the Sequencing Depth and Coverage Map. The abscissa represents the genome position ( unit: bp ), and the ordinate represents the sequencing depth ( unit: × ).The blue line in the figure shows the distribution of sequencing depth at different genomic locations, and the overall sequencing depth fluctuated between approximately 8 × and 550 ×.The key genomes and sequencing parameters are marked below the map: ( 1 ) the total length of the genome was 159,761 bp ; ( 2 ) the average sequencing depth was 193.27 × ; ( 3 ) the maximum sequencing depth was 550 × ; ( 4 ) the minimum sequencing depth was 8 ×.

1. **cis-splicing gene map**

**
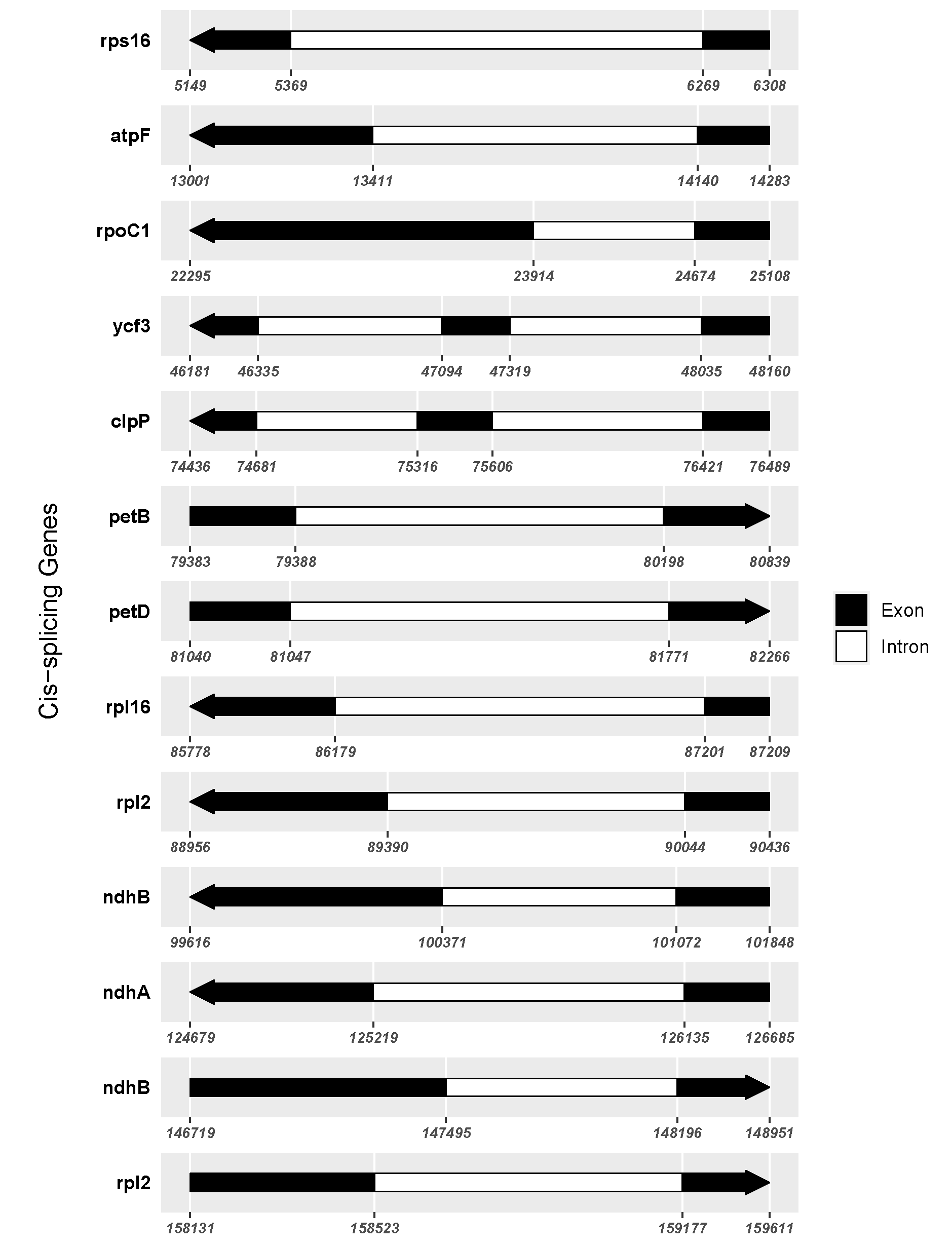
**

**Figure 2.** The map shows the gene structure of multiple Cis-splicing Genes , in which the black block represents the exon , the white block represents the intron , the arrow direction represents the transcription direction of the gene, and the number below is the position coordinates of the gene, showing the exon-intron composition and sequence position distribution characteristics of different genes ( such as rps16, atpF, and rpoC1). ).

1. **trans-splicing gene map.**


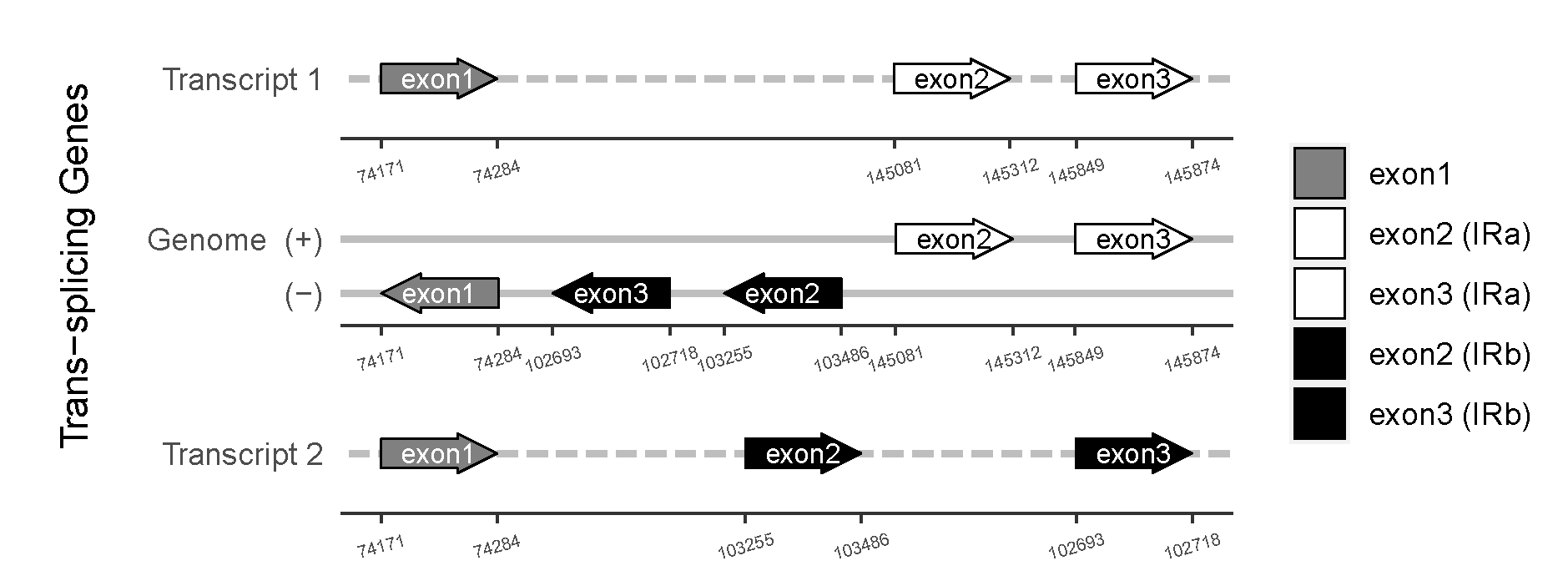


**Figure 3.** The figure shows the exon structure of the transcripts ( Transcript 1, 2 ) of the trans-splicing gene and the genome ( sense/antisense strand ). The exon type ( exon1, exon2 / 3 of IRa the IRb subtype ) is distinguished by color ( gray/white/black ), the sequence position is marked by coordinates, and the arrow direction represents the transcription/splicing direction. The core is: trans-splicing splices exons of different subtypes through the ' trans-genome chain ( sense/antisense ) ', resulting in two transcripts of IRa ( Transcript 1 ) and IRb ( Transcript 2 ). ' )

1. **coverage depth**


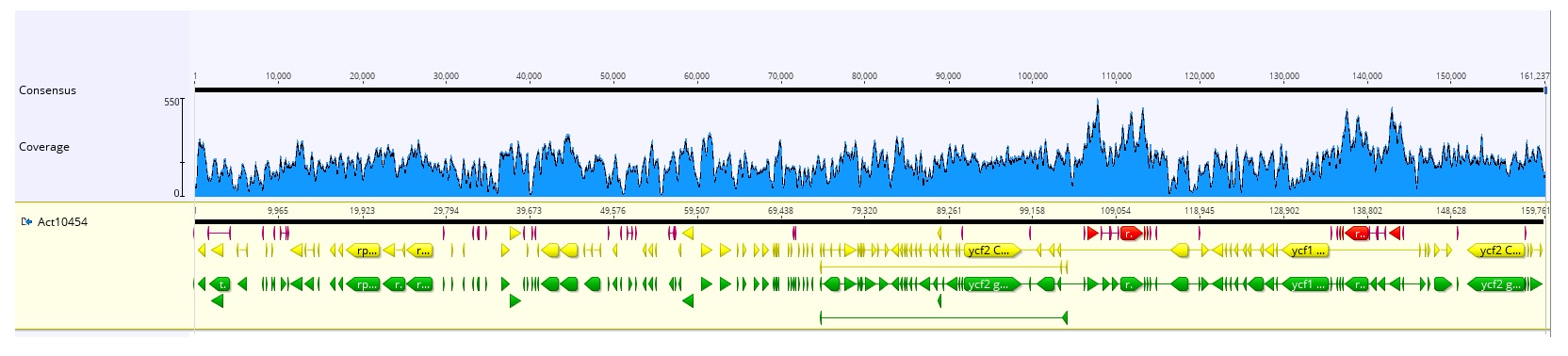


**Figure 4.** The figure shows the sequence coverage ( Coverage, blue curve, vertical axis 0.1-550 ) and gene annotation ( yellow and green arrows represent genes/elements in different directions, including annotations such as ' ycf1 ', ' ycf2 ', etc. ) of the Act10454 genome region ( horizontal axis from 1 to approximately 159,761 base pairs ). The coverage curve reflects the fluctuation in sequencing depth. The gene annotation layer presents the position and direction of the gene/functional elements ( such as reading frame, repeat sequence, etc. ) in the corresponding region of Act10454, which can be used to analyze the sequencing coverage characteristics and gene structure distribution of the genome region.
